# Supplementary material for: COZOID: contact zone identifier for visual analysis of protein-protein interactions
Source: BMC Bioinformatics. 2018 Apr 6;19:125. doi: 10.1186/s12859-018-2113-6 (PMC5889581; doi:10.1186/s12859-018-2113-6)
Supplement: Supplementary file 2 — Software build. Executable binary file of the software tool. (ZIP 118,784 kb) [file 12859_2018_2113_MOESM2_ESM.zip › cozoid/java/docs/beansbinding-1.2.1-doc.zip!/org/jdesktop/el/PropertyNotFoundException.html]

PropertyNotFoundException (Beans Binding)


---


|  |  |  |
| --- | --- | --- |
| **Overview** \| **Package** \| **Class** \| **Use** \| **Tree** \| **Deprecated** \| **Index** \| **Help** | |  |
| **PREV CLASS**   **NEXT CLASS** | **FRAMES**    **NO FRAMES**     **All Classes** |  |
| SUMMARY: NESTED \| FIELD \| CONSTR \| METHOD | DETAIL: FIELD \| CONSTR \| METHOD |  |


---


## org.jdesktop.el Class PropertyNotFoundException

```
java.lang.Object
  java.lang.Throwable
      java.lang.Exception
          java.lang.RuntimeException
              org.jdesktop.el.ELException
                  org.jdesktop.el.PropertyNotFoundException
```

**All Implemented Interfaces:**: java.io.Serializable

---

``` public class PropertyNotFoundException extends ELException ```

Thrown when a property could not be found while evaluating a
`ValueExpression` or `MethodExpression`.

For example, this could be triggered by an index out of bounds
while setting an array value, or by an unreadable property while
getting the value of a JavaBeans property.

**Since:**
:   JSP 2.1

**See Also:**: Serialized Form

---

**Constructor Summary**

`PropertyNotFoundException()`
  
          Creates a `PropertyNotFoundException` with no detail message.

`PropertyNotFoundException(java.lang.String message)`
  
          Creates a `PropertyNotFoundException` with the provided
detail message.

`PropertyNotFoundException(java.lang.String pMessage,
java.lang.Throwable pRootCause)`
  
          Creates a `PropertyNotFoundException` with the given detail
message and root cause.

`PropertyNotFoundException(java.lang.Throwable exception)`
  
          Creates a `PropertyNotFoundException` with the given root
cause.


**Method Summary**

**Methods inherited from class java.lang.Throwable**

`fillInStackTrace, getCause, getLocalizedMessage, getMessage, getStackTrace, initCause, printStackTrace, printStackTrace, printStackTrace, setStackTrace, toString`

**Methods inherited from class java.lang.Object**

`clone, equals, finalize, getClass, hashCode, notify, notifyAll, wait, wait, wait`

**Constructor Detail**

### PropertyNotFoundException

```
public PropertyNotFoundException()
```

:   Creates a `PropertyNotFoundException` with no detail message.

---


### PropertyNotFoundException

```
public PropertyNotFoundException(java.lang.String message)
```

:   Creates a `PropertyNotFoundException` with the provided
    detail message.

    **Parameters:**: `message` - the detail message

---


### PropertyNotFoundException

```
public PropertyNotFoundException(java.lang.Throwable exception)
```

:   Creates a `PropertyNotFoundException` with the given root
    cause.

    **Parameters:**: `exception` - the originating cause of this exception

---


### PropertyNotFoundException

```
public PropertyNotFoundException(java.lang.String pMessage,
                                 java.lang.Throwable pRootCause)
```

:   Creates a `PropertyNotFoundException` with the given detail
    message and root cause.

    **Parameters:**: `pMessage` - the detail message: `pRootCause` - the originating cause of this exception


---


|  |  |  |
| --- | --- | --- |
| **Overview** \| **Package** \| **Class** \| **Use** \| **Tree** \| **Deprecated** \| **Index** \| **Help** | |  |
| **PREV CLASS**   **NEXT CLASS** | **FRAMES**    **NO FRAMES**     **All Classes** |  |
| SUMMARY: NESTED \| FIELD \| CONSTR \| METHOD | DETAIL: FIELD \| CONSTR \| METHOD |  |


---
